# Hepatoprotective effect of botanical drug formula on high-fat diet-induced non-alcoholic fatty liver disease by inhibiting lipogenesis and promoting anti-oxidation

**DOI:** 10.3389/fphar.2022.1026912

**Published:** 2022-11-24

**Authors:** De-Shan Ning, Yu-Ju Chen, Chien-Ju Lin, Ching-Chiung Wang, Hong-Wei Zhao, Kun-Teng Wang, Ming-Chung Lee, Lemmuel L. Tayo, Wan-Chun Chiu, Chiu-Li Yeh, Chia-Jung Lee

**Affiliations:** ^1^ Infinitus (China) Company Ltd., Guangzhou, China; ^2^ Ph.D. Program in Clinical Drug Development of Herbal Medicine, Taipei Medical University, Taipei, Taiwan; ^3^ School of Pharmacy, College of Pharmacy, Kaohsiung Medical University, Kaohsiung, Taiwan; ^4^ Graduate Institute of Pharmacognosy, Taipei Medical University, Taipei, Taiwan; ^5^ School of Pharmacy, Taipei Medical University, Taipei, Taiwan; ^6^ Traditional Herbal Medicine Research Center, Taipei Medical University Hospital, Taipei, Taiwan; ^7^ Herbiotek Co., Ltd., New Taipei City, Taiwan; ^8^ School of Chemical, Biological Materials Science and Engineering, Mapúa University, Manila, Philippines; ^9^ School of Nutrition and Health Sciences, College of Nutrition, Taipei Medical University, Taipei, Taiwan; ^10^ Department of Nutrition, Wan Fang Hospital, Taipei Medical University, Taipei, Taiwan

**Keywords:** non-alcoholic fatty liver disease (NAFLD), network pharmacology, herb-based supplements, puerarin, AMPK pathway, anti-oxidation

## Abstract

With the prevalence of obesity and other components of metabolic syndrome, Non-alcoholic fatty liver disease (NAFLD) has become increasingly common. In recent years, much attention has been paid to various plant sources, hoping to find a treatment for NAFLD in plants. The Livsooth authentic herbal formula (LAH, 樂悠本草), a botanical drug formula combined with Puerariae lobatae radix, Lonicerae japonicae flos, Hoveniae semen, and Siraitiae fructus. This study used a network pharmacology approach to predict the potential mechanisms of LAH against NAFLD. Gene Ontology (GO) and KEGG pathway enrichment analyses have identified potential biochemical and signaling pathways. Subsequently, the potential mechanism of action of LAH on NAFLD predicted by network pharmacology analysis was validated in a high-fat diet (HFD)-induced NAFLD model in C57BL/6 mice. Our results demonstrated that LAH ameliorated hepatocyte steatosis in liver tissue by activating the AMPK pathway and decreasing serum triglycerides, low-density lipoprotein, glucose, and cholesterol. Besides, LAH increased the hepatic antioxidant enzymes activities, suggested that LAH improved oxidative stress markers in HFD induced NAFLD mice. *In vitro* experiments confirmed that the active component of LAH, puerarin, regulates lipid accumulation through the AMPK pathway. In conclusion, our study shows that network pharmacology predictions are consistent with experimental validation. LAH can be a candidate supplement for the prevention of NAFLD.

## Introduction

Two In the past few decades, liver disease has become one of the leading causes of death worldwide. By 2010, it was estimated that approximately 4% of deaths worldwide were due to liver diseases, such as liver cancer and cirrhosis. Non-alcoholic fatty liver disease (NAFLD) is the main cause of cirrhosis, including a variety of pathological liver diseases, which are characterized by hyperlipidemia, inflammation, and fibrosis (steatohepatitis and steatosis, respectively). With the prevalence of obesity and other components of metabolic syndrome, NAFLD has become increasingly common (Byass, 2014; Brunt et al., 2015; Rinella, 2015). The steps of developing NAFLD includes liver triglyceride (TG) accumulation to develop into non-alcoholic steatohepatitis (NASH) followed by oxidative stress, autophagy, and inflammation, causing further damage. Overweight or obese individuals are prone to insulin resistance which can negate insulin signaling, increase lipolysis of adipose tissue in excess free fatty acids (FFAs) and induction fatty tissue inflammation (Eigentler et al., 2020; Stefan, 2020). Along with the hepatic lipid accumulation, the high levels of FFA, cholesterol, and lipid metabolites present in the liver induce lipotoxicity. Furthermore, free fatty acids increase the risk of oxidative stress and produce reactive oxygen species (ROS). Thus, research on ways to improve the clinical outcome of fatty liver disease is critical. Livsooth authentic herbal formula (LAH, 樂悠本草) consists of four botanical drugs, *Pueraria montana* var. *lobata* (Willd.) Maesen and S.M.Almeida ex Sanjappa & Predeep [Leguminosae; Puerariae lobatae radix], *Lonicera japonica* Thunb. [Caprifoliaceae; Lonicerae japonicae flos], *Hovenia dulcis* Thunb. [Rhamnaceae; Hoveniae semen] and *Siraitia grosvenorii* (Swingle) C.Jeffrey ex A.M.Lu & Zhi Y.Zhang [Cucurbitaceae; Siraitiae fructus]. Puerariae lobatae radix is a very useful Traditional Chinese medicine (TCM) for relieving head and neck pain, reducing fever, clearing measles, promoting body fluid, and relieving diarrhea (Ehrman et al., 2007; Zhao et al., 2016). Modern pharmacological investigations have shown that this botanical cocktail has extensive biological activities such as hepatoprotective (Zhou et al., 2018; Sun et al., 2019), antioxidant (Jiang et al., 2005), anti-inflammatory (Jin et al., 2012) and anti-cancer (Ahn et al., 2019). Lonicerae japonicae flos is well-known as clearing heat and detoxifying. Many studies have described the anti-inflammatory (Yang et al., 2019a; Yang et al., 2021) and antiviral activities (Zhou et al., 2017) of honeysuckle. It inhibits oxidative damage to hepatocytes by increasing the activity of antioxidant enzymes which scavenge reactive oxygen species and other free radicals (Gao et al., 2018). Hoveniae semen has been used in the treatment of ethanol-induced liver disease for centuries. Hoveniae semen could modulate abnormalities of the gut-liver axis and inhibits TLR4-associated inflammatory mediator activation to exert its hepatoprotective properties (Cho et al., 2016; Jeong et al., 2019; Qiu et al., 2019). Siraitiae fructus is a medicinal and edible plant with various health-promoting properties. It serves as a promising antiglycative agent against diabetic complications by inhibiting protein glycation and glycoxidation (Chen et al., 2011; Liu et al., 2018). These four botanical drugs have been reported to have protective effects on the liver, suggesting a potential therapeutic effect on NAFLD. However, no scientific experiments and clinical trials have been conducted to verify its effectiveness or to explore its potential mechanism against NAFLD.

In recent years, much attention has been paid to various plant sources, hoping to find a treatment for NAFLD in plants. TCM has been used in China and other countries for thousands of years. A specific and basic feature of TCM is the use of formulas containing several botanical drugs to ameliorate the abnormal symptoms associated with a particular disease (Guo et al., 2017). A wide variety of botanical remedies have traditionally been used to treat NAFLD and metabolic syndromes. Having multiple biological targets and multiple therapeutic mechanisms are characteristic features of TCM formula, and these substances may provide potential therapeutic effects on the multifactorial NAFLD (Xu et al., 2020; Fan et al., 2021). Network pharmacology is a new approach to predict or reveal complex mechanisms of TCM formula, which updates the research model from the current “one target, one drug” model to a new “network-target, multiple-component” model (Zhang et al., 2019). By providing a detailed composite target and target pathway network, it is helpful to evaluate the integrity, systemic and interaction of TCM. Therefore, the application of network pharmacology to TCM compounds can be used to explain the mechanism of action of TCM, discover medicinal active ingredients, and provide new ideas for the development and research of new drugs.

**FIGURE 1:**
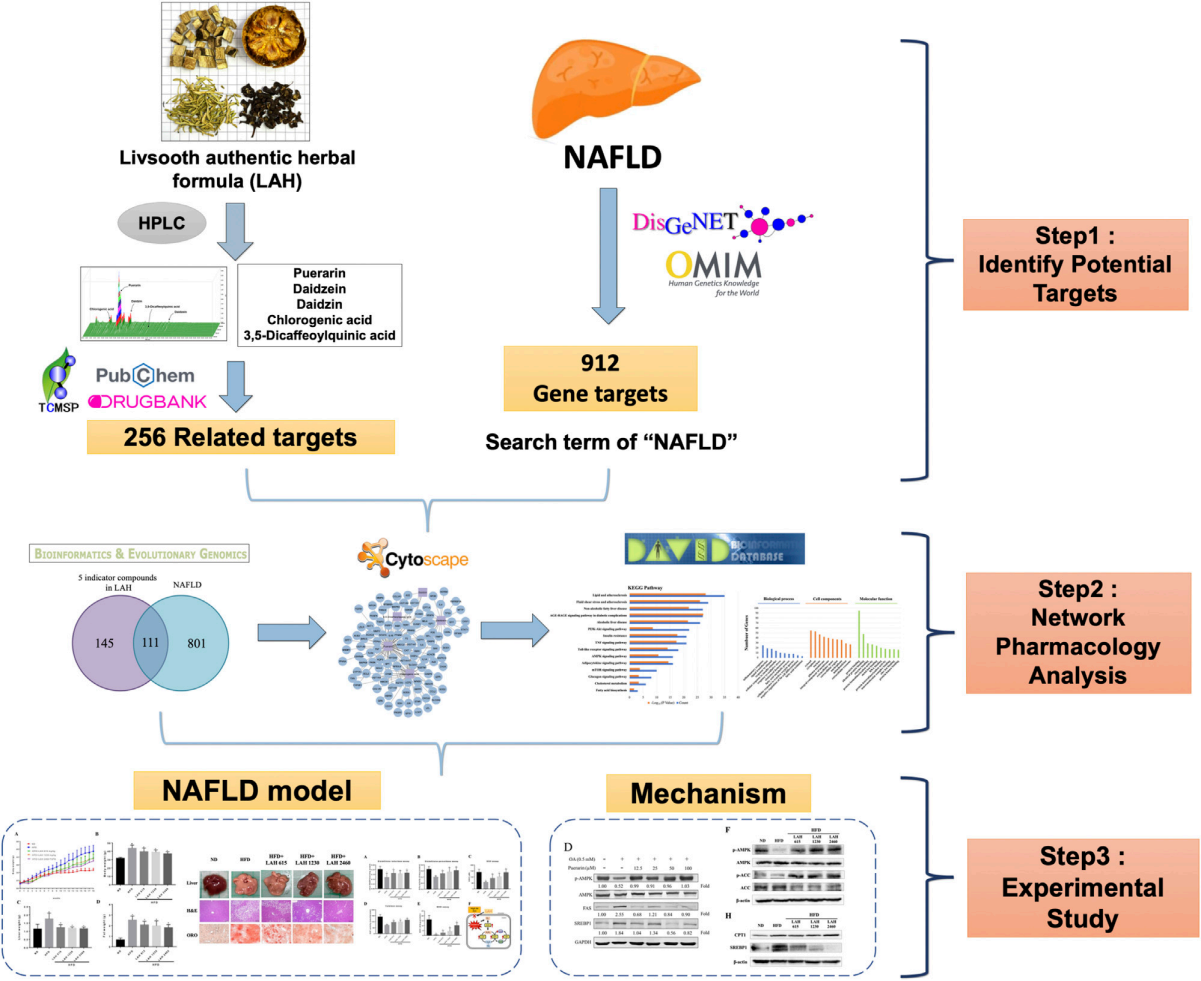
Flowchart of a network pharmacology study of LAH in the treatment of NAFLD.

In this study, we used a network pharmacology approach to predict potential pathways of LAH to NAFLD. In addition, a mouse model of NAFLD was established by feeding a high-fat diet (HFD) and used to verify whether the effect and mechanism of LAH on NAFLD *in vivo* was as predicted by the network pharmacology approach. The detailed research flowchart is shown in Figure 1.

## Materials and methods

### Preparation of Livsooth authentic herbal mix

The Livsooth authentic herbal formula (LAH) prescription from Infinitus Pharmaceutical combines *Pueraria montana* var. *lobata* (Willd.) Maesen and S.M.Almeida ex Sanjappa & Predeep [Leguminosae; Puerariae lobatae radix], *Lonicera japonica* Thunb. [Caprifoliaceae; Lonicerae japonicae flos], *Hovenia dulcis* Thunb. [Rhamnaceae; Hoveniae semen] and *Siraitia grosvenorii* (Swingle) C.Jeffrey ex A.M.Lu & Zhi Y.Zhang [Cucurbitaceae; Siraitiae fructus]. The medicinal materials were authenticated by a non-profit organization, the Brion Research Institute of Taiwan. Voucher specimens (No. PR-20180001 for Puerariae lobatae radix, No. LJF-20180001 for Lonicerae japonicae flos, No. HS-20180001 for Hoveniae semen and No. SF-20180001 for Siraitiae fructus) was deposited at the College of Pharmacy, Taipei Medical University. Botanical drugs of LAH (Puerariae lobatae radix: Lonicerae japonicae flos: Hoveniae semen: Siraitiae fructus = 48: 26: 8: 1) were extracted with 10-fold boiling water for 2 h, twice. Combined extraction solution was removed excess water to the solid content about 60% by vacuum concentration. The LAH granules were obtained by 80°C dry granulation. The above extraction procedure was under Good Manufacturing Practice (GMP) in China.

### High-performance liquid chromatography sample preparation

For high-performance liquid chromatography (HPLC) analysis, a 0.5 g sample was extracted using 20 ml of 70% methanol through ultrasonic oscillation at 25°C for 20 min. The sample was then filtered through a 0.45 μm syringe filter.

### High-performance liquid chromatography analysis of marker substances in LAH

The Waters HPLC system (Milford, Massachusetts, United States) was comprised of Waters 600 pump system, Waters 2996 Photodiode array detector, Waters 717 plus Autosampler, and Sugai U-620 Column oven (Wakayama City, Japan). Cosmosil 5C18-MS-II reversed phase column (5 μm, 4.6 × 250 mm, Nacalai tesque, Japan) equipped with Lichrospher RP-18 end-capped guard column (5 μm, 4.0 × 10 mm, Merck, Germany) was used as the stationary phase. The gradient elution was composed of eluents A, B, and C (A: H_2_O/KH_2_PO_4_/10% H_3_PO_4_ = 1000 ml/2.72 g/1 ml; B: Acetonitrile; C: H_2_O) according to the following profile: 0–30 min, 90%–75% A and 10%–25% B; 30–40 min, 75%–65% A and 25%–35% B; 40–55 min, 65%–0% A, 35%–75% B and 0%–25% C; 55–60 min, 75%–10% B and 25%–90% C; 60–65 min, 0%–90% A, 10% B and 90%–0% C. The gradient elution was used for 3D fingerprint analysis and quantification of puerarin (250 nm), daidzin (250 nm), daidzein (250 nm), chlorogenic acid (320 nm) and 3,5-dicaffeoylquinic acid (325 nm). The flow rate was 1 ml/min, and the column temperature was maintained at 35°C.

### Predicting the mechanism of action of the five major components of LAH in non-alcoholic fatty liver disease based on network pharmacology

This study recruited compound names which were used as keywords in TCMSP (http://tcmspw.com/tcmsp.php), DrugBank (https://go.drugbank.com/) and PubChem (https://pubchem.ncbi.nlm.nih). gov/) database search components for gene targets, standardized using UniProt KB database while deleting duplicates to obtain 256 potential targets. A list of NAFLD-related targets was collected from the OMIM (https://omim.org/) (Amberger et al., 2014; Zhang et al., 2020) and DisGeNET (https://www.disgenet.org/) (Piñero et al., 2016) repositories using the search term “non-alcoholic fatty liver disease” or “NAFLD” or “fatty liver, nonalcoholic” or “liver, nonalcoholic fatty” or “livers, nonalcoholic fatty” or “nonalcoholic fatty liver” or “nonalcoholic steatohepatitis” or “steatohepatitis, nonalcoholic,” delete duplicates to obtain 912 gene targets. To understand the mechanism of LAH in NAFLD, network analysis was performed. Component-Target network was established and visualized by Cytoscape 3.9.1 software. For predicting the mechanism of action of LAH in NAFLD, putative targets were loaded into DAVID (https://david.ncifcrf.gov/) for enrichment analyses in KEGG (Kyoto Encyclopedia of Genes and Genomes) pathways and the GO (Gene Ontology) enrichments. Adjusted *p*-value ≤ 0.05 and count ≥2 was chosen in functional annotation clustering.

### Animals and experimental protocol

Five-week-old male C57BL/6 mice were procured from the National Laboratory Animal Center in Taiwan and kept on a 12-h light/12-h dark cycle at 21 ± 2°C with food and water ad libitum. C57BL/6 mice were randomized into a ND group: fed with chow diet (ND) and oral administration ddH_2_O, a high-fat diet group (HFD) fed a commercial diet containing 60% fat and oral administration ddH_2_O (High-fat diet (No. 58Y1) was purchased from TestDiet, Inc.), and three LAH groups: fed with a high-fat diet and oral administration of LAH at doses of 615 mg/kg, 1230 mg/kg, and 2460 mg/kg; each group n = 10. All experiments were carried out for 18 weeks, and body weight and obesity-related biomarkers were periodically recorded. At the end of the experiment, the mice were euthanized with CO_2_ after fasting for 12 h, and then blood and tissue samples were collected. The animal use protocol was reviewed and approved by the Institutional Animal Care and Use Committee or Panel (IACUC/IACUP), Taipei Medical University (IACUC Approval no. LAC-2019-0070). All methods involved in the animal experiments were performed in accordance with the relevant guidelines and regulations.

### Histological and oil red O analysis

Liver tissue was fixed in 4% formaldehyde and embedded in paraffin. All tissues were sliced into 10-μm sections and stained with hematoxylin and eosin (H&E). OCT-embedded frozen liver sections were stained with Oil Red O. Briefly, liver sections were fixed with 4% formaldehyde at room temperature for 15 min. After fixation, the cells were washed three times with phosphate-buffered saline (PBS) and stained with 0.6% (w/v) Oil Red O solution for 15 min at room temperature. The sections were quickly washed with 60% isopropanol one time, washed three times with PBS, and photographed using a Nikon microscope.

### Serum biochemical analysis

Blood was obtained from the heart after the end of the 18-weeks, centrifuged blood samples at 13,000 rpm for 10 min to separate the serum. Serum levels of glucose (GLU), triglyceride (TRIG), total cholesterol (CHOL), alanine aminotransferase (ALT), aspartate aminotransferase (AST), and albumin (ALB) were analyzed using VetTest 8008 (IDEXX Lab Inc., Westbrook, ME, United States) according to the manufacturer’s protocol.

### Western blot assay

Tissues and cells were lysed in ice-cold RIPA buffer, and protein quantification was performed using the Bradford assay (Bio-Rad Laboratories, Hercules, CA, United States). Lysates were electrophoresed on 8%–10% SDS-PAGE gels and transferred to an immunoblot polyvinylidene difluoride (PVDF) membrane using a semi-dry transfer system (Bio-Rad, Hercules, CA, United States). The PVDF membranes were blocked with 5% BSA at room temperature for 1 h and incubated at 4°C overnight with the following primary antibodies. These membranes were several washed with TBST buffer, secondary antibodies and chemiluminescent detection reagents were applied sequentially. Primary specific antibodies included β-actin (Santa Cruz Biotechnology, CA, United States), CPT1, SREBP1, phospho-AMPK, AMPK, and phospho-ACC (Cell Signaling Technology, Danvers, MA, United States). Western blots were quantified by ImageJ.

### Assay for oxidative stress-related parameters in liver

Liver tissue homogenates were prepared by high-speed stirring of liver tissue in ice-cold buffer (0.25 M sucrose, 1 mM EDTA, 10 mM Tris-HCl), then centrifuged for 10 min (4500 × g, 4°C), and the liver cytoplasm separated and stored at −80°C for further analysis. Superoxide dismutase (SOD, Rondox SD125, Antrim, United Kingdom), glutathione reductase (GR, Randox GR 2368, Goldberg, DM), glutathione peroxidase (GPx, Randox RS 504, Paglia), glutathione (GSH, Catalog No.703002), catalase (CAT, Cayman No.707002), LDL and HDL (Randox CH201, Antrim, United Kingdom), and triglyceride (TRIG, TR213, Antrim, United Kingdom) levels in the liver homogenates were determined using biochemical kits.

### Cell culture and fatty liver cells treatment

The HepG2 hepatocyte cell line was purchased from the Bioresource Collection and Research Center (BCRC, Taiwan) and cultured at 37°C in a 5% CO_2_ atmosphere in DMEM medium supplemented with 1% penicillin/streptomycin, 1% l-glutamine and 10% fetal bovine serum (FBS). HepG2 cells were incubated with 0.5 mM oleic acid (St. Louis, MO, United States) and co-treated with LAH (12.5–400 μg/ml) to stimulate lipid accumulation for 24 h then evaluate the mechanism of lipid metabolism.

### Cell viability assay

HepG2 cells were incubated with various concentrations of LAH for 24 h to determine cell viability using MTT solution (Sigma). Next, the culture plates were treated with DMSO to evaluate cell viability. Absorbance was then measured at 600 nm using an ELISA plate reader (BioTek, Winooski, VT, United States).

### Oil red O staining

HepG2 cells were seeded and grown in 48-well plates and co-treated with 0.5 mM oleic acid and LAH (12.5–400 μg/ml) for 24 h. Cells were washed three times with PBS and fixed with 4% formaldehyde at room temperature for 1 h. After fixation, cells were stained with Oil Red O solution for 1 h and washed with 60% isopropanol to detect oil droplets. For quantitative analysis of cellular lipids, Oil Red O was eluted with 100% isopropanol and quantified spectrophotometrically at 520 nm using an ELISA plate reader (BioTek, Winooski, VT, United States).

### Statistical analysis

All data are presented as mean ± SD. The difference between multiple groups was analyzed using one-way analysis of variance (ANOVA) followed by Tukey’s multiple comparison test. A *p* value <0.05 was considered statistically significant. All statistical analyses were using GraphPad Prism Software version 7.0 (GraphPad Software Inc., San Diego, CA, United States).

## Results

### Analysis of the main components of LAH using high-performance liquid chromatography

**FIGURE 2:**
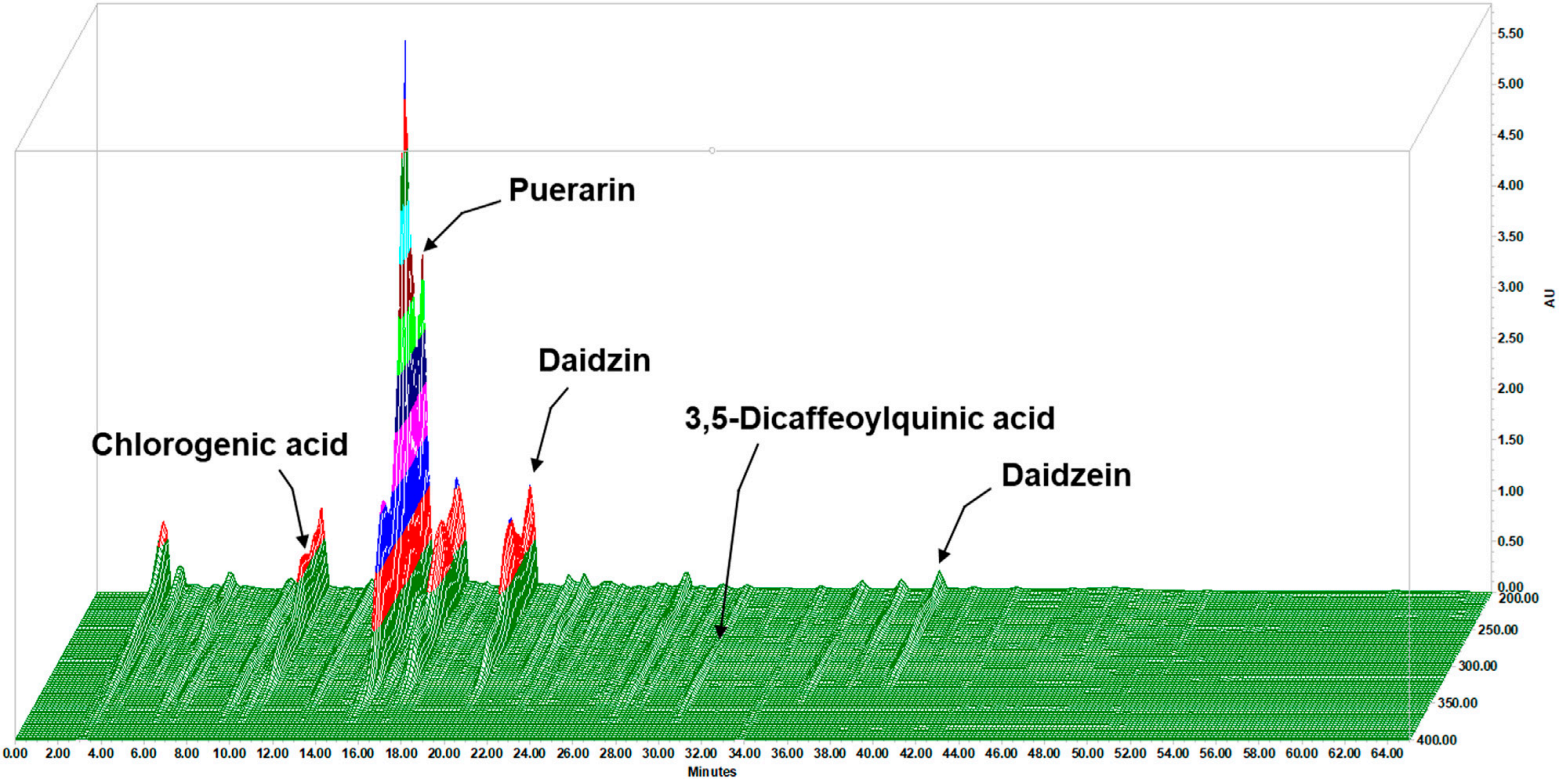
3D fingerprint analysis of the LAH by using high-performance liquid chromatography (HPLC).

The contents of the components in LAH were analyzed using the HPLC method. The HPLC chromate-graphic profile indicated that LAH contained chlorogenic acid, puerarin, daidzin, daidzein and 3,5-dicaffeoylquinic acid. The contents of the investigated analytes were as follows: chlorogenic acid for 3.87 mg/g, puerarin for 57.33 mg/g, daidzin for 14.82 mg/g, 3,5-dicaffeoylquinic acid for 0.46 mg/g and daidzein for 1.70 mg/g in LAH, as well as the peaks of retention times were at 11.36, 15.40, 20.31, 30.22, and 39.32 min, respectively (Figure 2).

### Network pharmacology analysis of LAH treating non-alcoholic fatty liver disease

**FIGURE 3:**
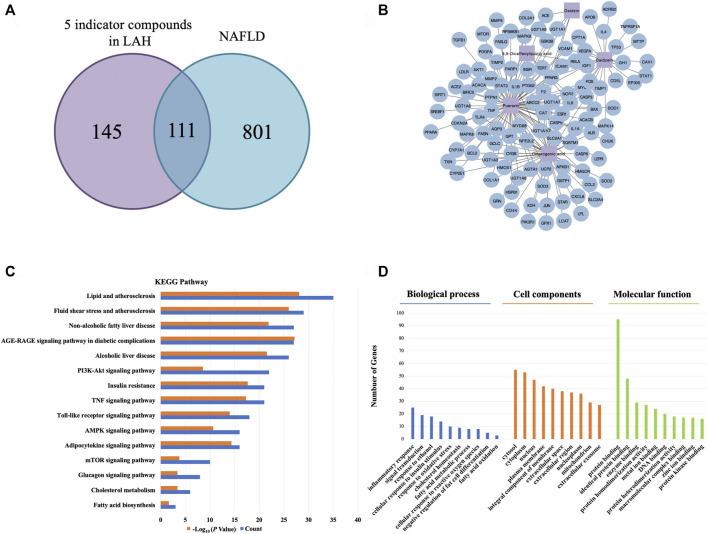
Network pharmacology analysis for LAH on NAFLD. **(A)** Venn diagrams of potential genes in LAH and NAFLD. **(B)** LAH Component-Target network. The purple square represents the 5 major components, and the blue circle represent the target genes. **(C)** The KEGG pathway enrichment analyses of 111 target proteins. **(D)** The GO enrichment analyses of 111 target proteins.

According to HPLC analysis, chlorogenic acid, puerarin, daidzin, 3,5-dicaffeoylquinic acid and daidzein are the major components in LAH. To further study the mechanism of action of LAH against NAFLD, it is crucial to understand the target genes of these 5 components. Network pharmacology provides an effective tool for the study of TCM pharmacology. A total of 256 potential targets were retrieved from the TCMSP, DrugBank and PubChem databases of LAH. Further, a total of 912 gene targets associated with NAFLD were retrieved from OMIM and DisGeNET databases. Using Venn diagram to analyze the gene targets of LAH and the gene targets related to NAFLD in the database, it was found that 111 targets overlapped, which were considered as potential targets of LAH against NAFLD (Figure 3A). To predict the underlying mechanism of LAH to NAFLD, these targets were constructed using Cytoscape to plot a Component-Target network (Figure 3B). To identify biological signatures of LAH-related targets of NAFLD, KEGG pathway and GO enrichment analysis was performed on the involved targets. The significantly enriched KEGG pathways of 111 putative targets contained lipid and atherosclerosis, non-alcoholic fatty liver disease, AMPK signaling pathway, PI3K-Akt signaling pathway, adipocytokine signaling pathway, etc, demonstrate the effect of LAH against NAFLD was closely related to lipogenesis and promoting anti-oxidation effect (Figure 3C). In terms of GO enrichment analysis, it was also shown that LAH may exert its ameliorating effect on NAFLD by regulating fatty acid metabolism and response to oxidative stress *via* protein binding, the identical protein binding and enzyme binding (Figure 3D).

### LAH reduces lipid accumulation in HepG2 cells

**FIGURE 4:**
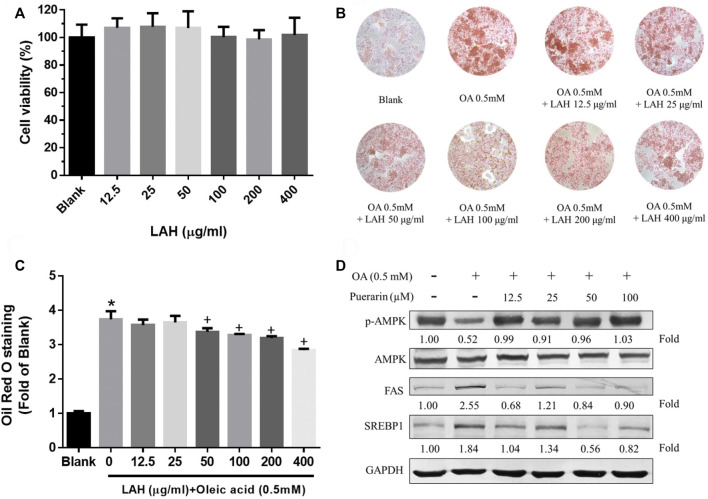
LAH and puerarin regulate OA-induced lipogenesis in HepG2 cells. **(A)** Cell viability of HepG2 cell treated with LAH, determined over 24 h using an MTT assay. **(B)** Cells were plated into 24well plate and treated with different doses of LAH in the presence of OA (0.5 mM) for 24 h. ORO staining of HepG2 cells after treatment with OA and LAH. **(C)** Quantitative lipid accumulation of Oil Red O contents at 520 nm. **(D)** Phosphorylated AMPK, AMPK, FA, and SREBP1 protein levels by western blot analysis. Protein expression was normalized to GAPDH. Values are expressed as mean ± SD (*n* = 3/group). *p < 0.05 compared with Blank group. + *p* < 0.05 compared with OA group.

We used the MTT assay to determine the cytotoxicity of LAH in HepG2 cells to investigate whether LAH regulates lipid metabolism in hepatocytes *in vitro*. There was no cell cytotoxicity in the range of 0–400 μg/ml (Figure 4A). To investigate the effect of LAH on hepatic lipid accumulation, cellular lipid deposits were induced by oleic acid (OA) in HepG2 cells. Oil red O staining confirmed that 0.5 mM OA increase lipid droplets accumulation in HepG2 cells, compared with the Blank group. LAH alleviated intracellular lipid droplets in a dose-dependent compared to OA induced HepG2 cells (Figures 4B,C).

### Effect of puerarin on lipogenesis-related proteins in the HepG2 cells

To prove puerarin is the major active component of LAH, we investigate its capability in regulating lipid accumulation, expression of lipogenesis-related proteins, phospho-AMPK, SREBP1, and FAS via western blot analyses. OA downregulated the protein expression levels of phospho-AMPK. The puerarin cotreatment was a significant increase phospho-AMPK protein level in HepG2 cells. The FAS, and SREBP1 protein expressions were found inhibited by puerarin, compared with OA group. (Figure 4D). Therefore, these results indicate that puerarin reduces lipid accumulation in OA-treated HepG2 cells by regulating AMPK/SREBP1 and lipogenesis synthesis pathway. It is suggested that puerarin can be used as an active indicator component of LAH.

### Effect of LAH on body weight, fat weight, liver weight and hepatic histological changes in high-fat diet fed mice

**FIGURE 5:**
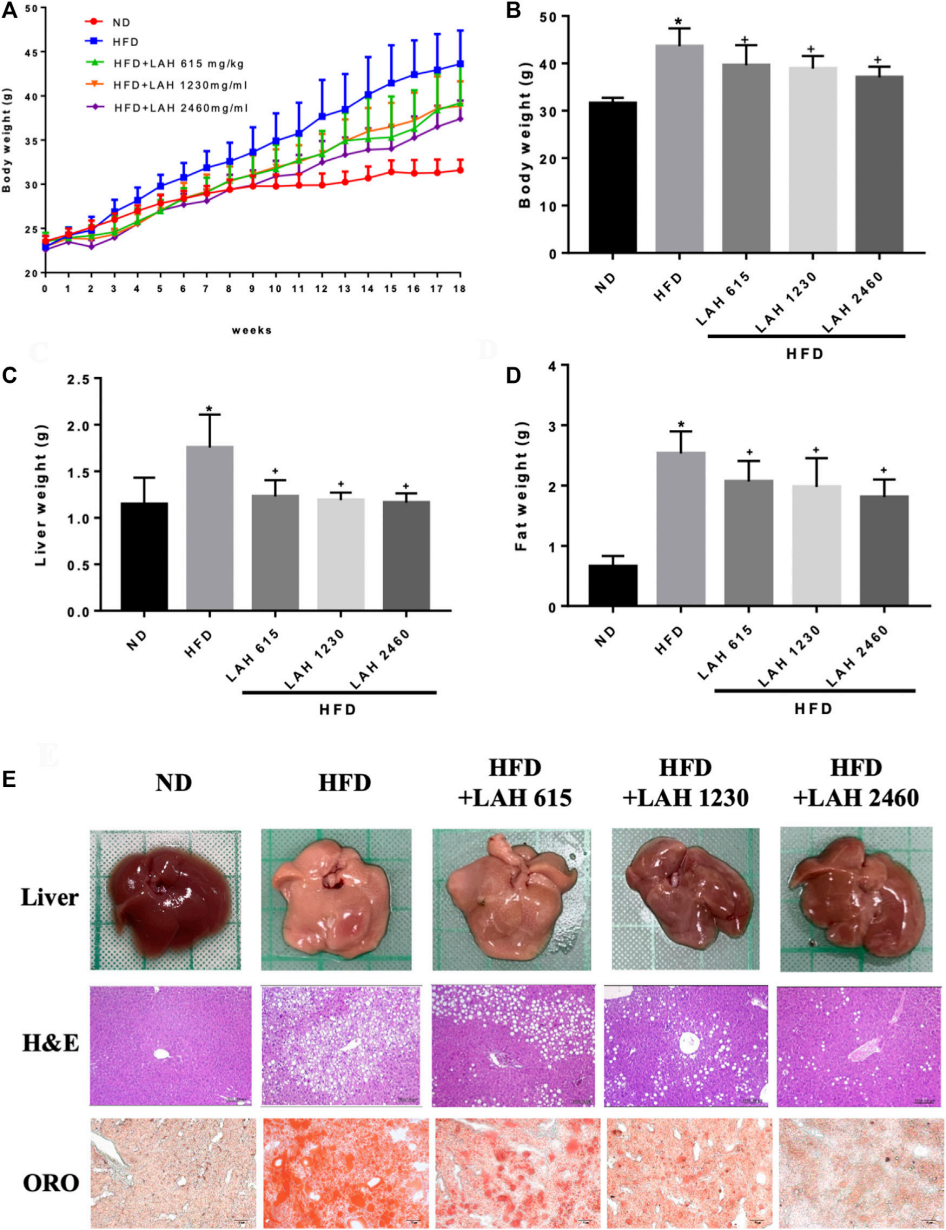
LAH ameliorated hepatic steatosis in HFD-induced mice. **(A)** Changes in body weight of mice fed either chow diet (ND), high-fat diet (HFD), or HFD containing 615 mg/kg, 1230 mg/kg, or 2460 mg/kg LAH extract for 18 weeks, **(B)** final body weight, **(C)** final liver and **(D)** inguinal adipose tissue weights, **(E)** Morphological photographs of livers. Histological analysis of liver sections stained with hematoxylin-eosin (H&E) staining and oil red O (100 × magnification). Values are expressed as mean ± SD (*n* = 10/group). *p < 0.05 compared with ND group. + *p* < 0.05 compared with HFD group.

Before administration of LAH and HFD, the body weight values were not different between the groups. After feeding with a high-fat diet, the body weight of the HFD group was significantly higher than that of the ND group. In LAH-treated groups were significantly decrease the body weight compared with the HFD group (Figures 5A,B). The weight of the liver and fat presented the same result. Liver weight in all LAH groups were significantly lower than the HFD group and close to the ND group. Fat weights were significantly lower in all LAH groups than in the HFD group (Figures 5C,D). In addition, to determine the histological effects of LAH on hepatic steatosis, liver histological slices used H&E staining to detect lipid accumulation and hepatocyte damage. Significant histological abnormalities, including fat deposition in hepatocytes and inflammatory cell infiltration, were observed in the HFD group liver tissue, as compared with the ND group. However, these changes in pathology were alleviated in LAH-treated (615 mg/kg, 1230 mg/kg, or 2460 mg/kg) groups. Furthermore, Oil Red O staining observed that the lipid deposition in hepatocytes of the HFD group was significantly increased compared to the ND group; the deposition of lipid droplets in the hepatocytes of the LAH group was significantly decreased (Figure 5E).

### Effects of LAH on the biochemical parameters

**FIGURE 6:**
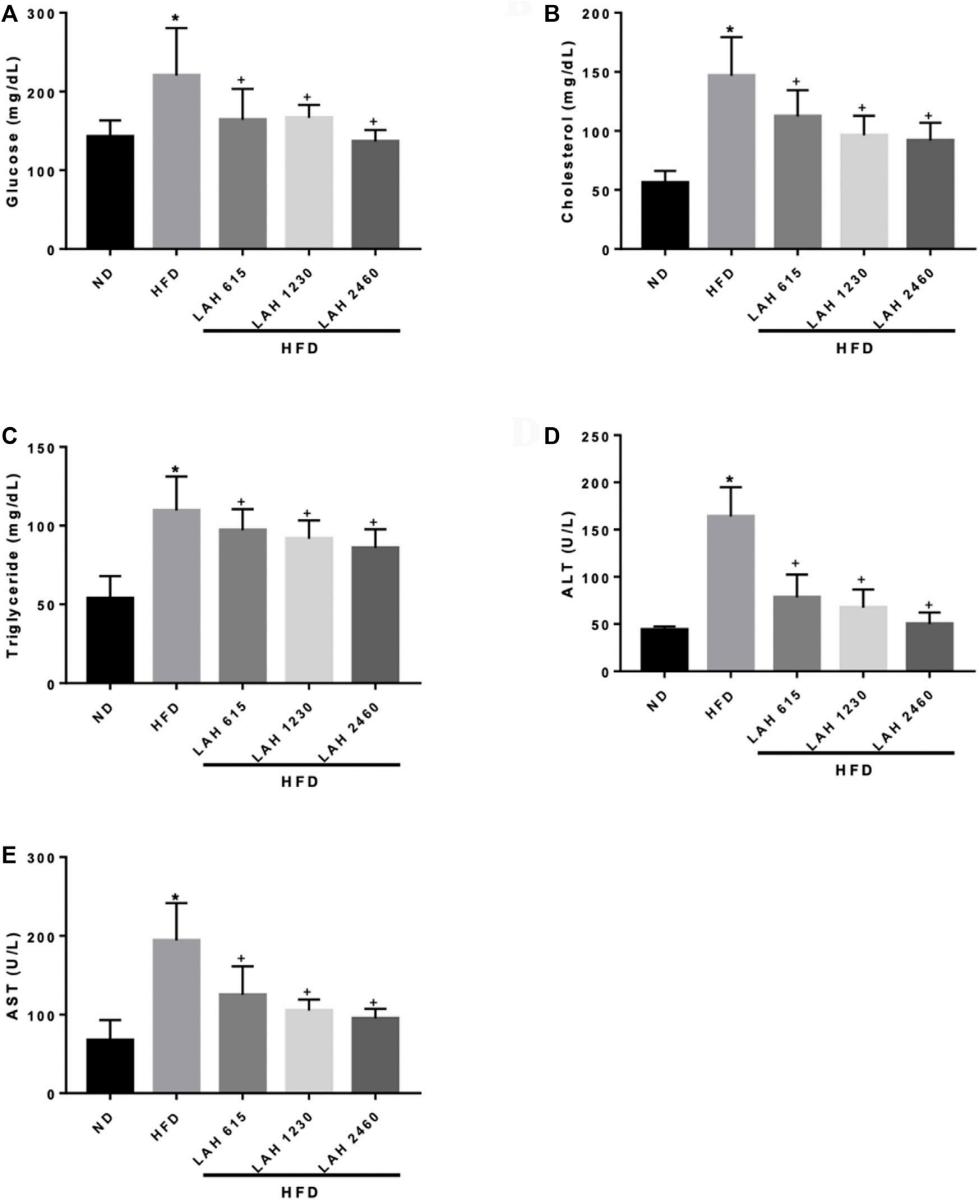
Serum biochemical analysis after 18 weeks of supplementation with LAH. Biochemical analysis of serum samples. Levels of **(A)** Fasting blood glucose, **(B)** CHOL, **(C)** TG, **(D)** ALT, and **(E)** AST were measured in the serum of mice in different experimental groups. Values are expressed as mean ± SD (*n* = 10/group). *p < 0.05 compared with ND group. + *p* < 0.05, compared with HFD group.

The levels of blood glucose, CHOL, TC, ALT, and AST in HFD group were higher than those in ND group. This means that HFD-induced mice develop hyperglycemia, dyslipidemia, and liver damage. The results showed that LAH treatment (615 mg/kg, 1230 mg/kg, or 2460 mg/kg) significantly reduced HFD-induced increases of blood glucose, CHOL, TC, ALT, and AST levels in a dose-dependent manner. These results suggest that LAH may prevent HFD-induced hyperglycemia, dyslipidemia, and liver damage (Figures 6A–E).

### Effects of LAH on the hepatic lipid accumulation

**FIGURE 7:**
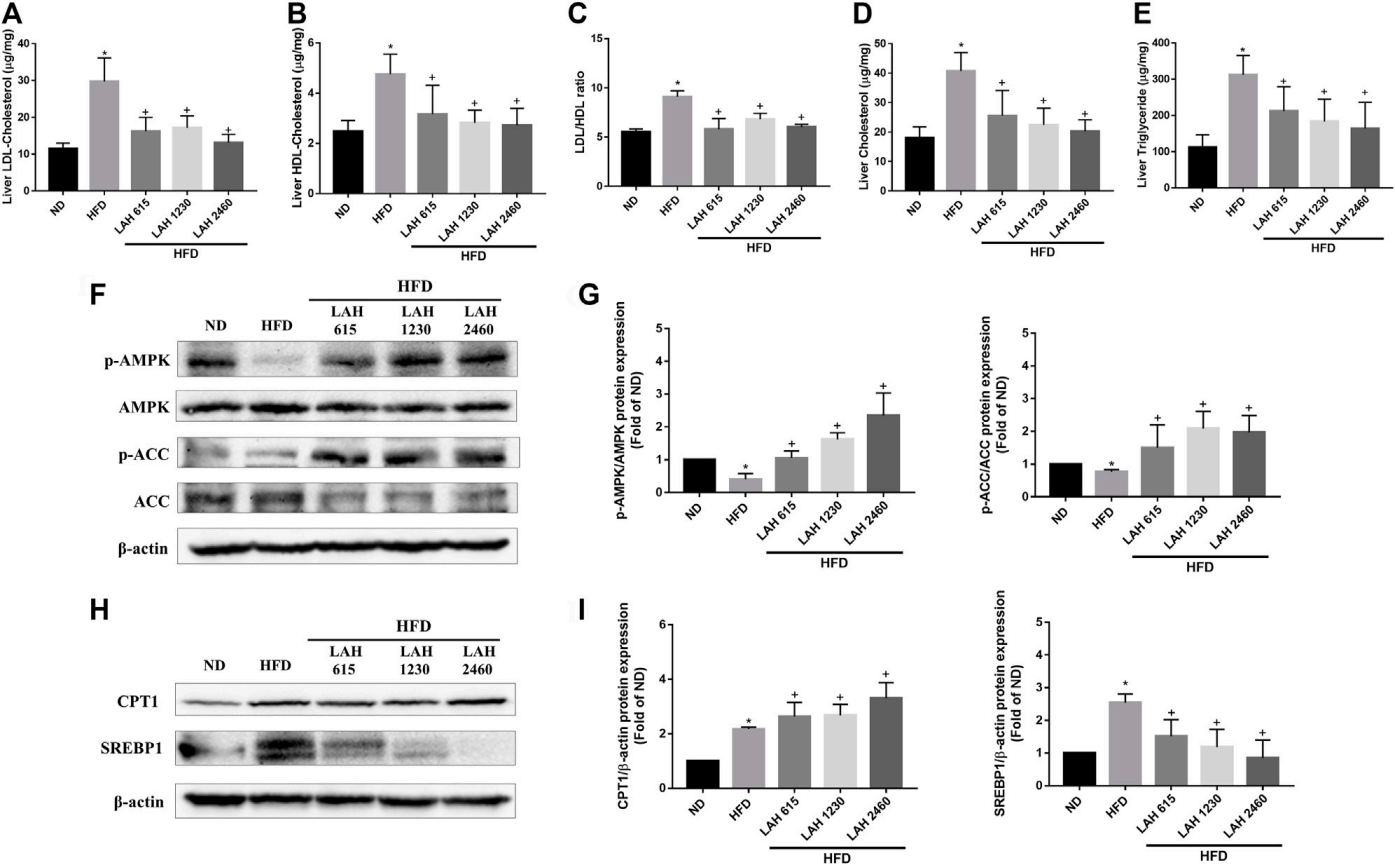
LAH treatment ameliorates HFD-induced hepatic lipids accumulation via AMPK-SREBP1 pathway. Liver content of **(A)** LDL, **(B)** HDL, **(C)** LDL/HDL ratio, **(D)** CHOL, and **(E)** TG in the ND, HFD, or HFD containing 615 mg/kg, 1230 mg/kg, and 2460 mg/kg LAH extract groups are shown (***n*** = 10 per group). **(F)** Phosphorylated AMPK, AMPK, Phosphorylated ACC, ACC protein levels by western blot analysis. **(G)** Phospho-AMPK and phospho-ACC protein expression was normalized to total AMPK and ACC, respectively. **(H)** CPT1, and SREBP-1 protein levels by western blot analysis. **(I)** CPT1 and SREBP1 protein expression was normalized to β-actin. Values are expressed as mean ± SD (n = 3/group). *p < 0.05, compared with ND group. + *p* < 0.05 compared with HFD group.

We next investigated the effect of LAH on hepatic lipid accumulation. Mice fed the HFD showed markedly increased LDL and HDL levels in the liver compared to the ND mice. The mice administered with LAH (615 mg/kg, 1230 mg/kg, or 2460 mg/kg) had significantly decreased LDL and HDL levels in the liver compared to the HFD mice (Figures 7A,B). In addition, the LDL-to-HDL ratio of HFD-fed mice was significantly higher than that of ND mice, and this ratio was markedly reduced by LAH treatment (Figure 7C). The levels of hepatic TC and TG in the HFD mice were significantly higher than those in the ND mice. In comparison to the HFD mice, hepatic TC and TG levels in the LAH mice were lower (Figures 7D,E). To investigate the molecular mechanisms, we evaluated protein expression during hepatic lipogenesis by Western blotting. LAH supplemented group caused increases in fatty acid oxidation-related protein expression as well as phospho-AMPK, phospho-ACC and CPT1 levels, compared to the HFD group (Figures 7F–I). Furthermore, SREBP1 protein levels increased significantly in the HFD group compared with the ND group. LAH treatment downregulated the SREBP1 protein expression compared with HFD group (Figures 7H,I). These results indicate that LAH can protect against lipid accumulation in the liver and enhance fatty acid oxidation by regulating AMPK-SREBP1 pathway.

### Anti-oxidative effects of LAH on liver tissue

**FIGURE 8:**
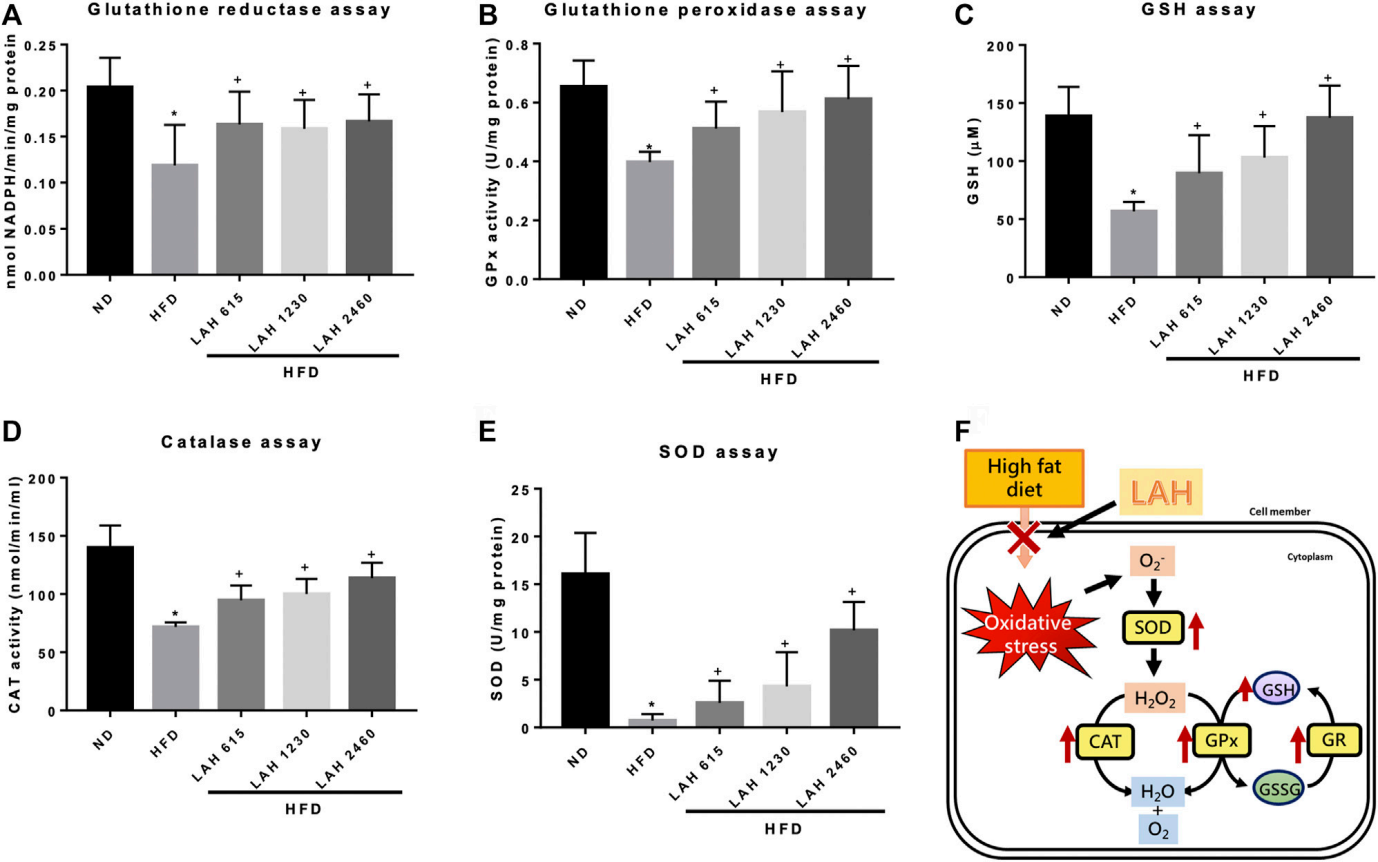
LAH attenuates hepatic oxidative stress and enhances antioxidant enzyme activity in HFD-mice. Levels of **(A)** Glutathione reductase (GR), **(B)** Glutathione peroxidase (GPx), **(C)** GSH, **(D)** CAT, and **(E)** SOD in liver tissue. **(F)** A schematic illustration of the antioxidant effect of LAH. Values are expressed as mean ± SD (*n* = 10/group). *p < 0.05 compared with ND group. + *p* < 0.05 compared with HFD group.

Excessive accumulation of fatty acids will increase the production of ROS. To understand the anti-oxidative effect of LAH on HFD-induced mouse liver tissues, we evaluated glutathione reductase (GR), glutathione peroxidase (GPx), GSH, CAT, and SOD (Figures 8A–E) levels. The activity of these enzymes was significantly reduced by the HFD. However, LAH treatment (615 mg/kg, 1230 mg/kg, or 2460 mg/kg) upregulates these enzymes activity which is like the ND group. The results suggest that LAH attenuates HFD-induced oxidative stress in hepatocytes by increasing the potential of antioxidant enzymes. (Figure 8F).

## Discussion

The essence of Chinese medicine lies in the use of formulas containing several botanical drugs to achieve better effects and improve abnormal symptoms associated with a specific disease. Previous studies have shown that TCM formulas have a protective effect against NAFLD (Yang et al., 2019b; Li et al., 2020). Puerariae lobatae radix, Lonicerae japonicae flos, Hoveniae semen, and Siraitiae fructus have been used to treat metabolic diseases. Recent research suggests that Puerariae lobatae radix improves glucose and lipid metabolism and exerts protective effects against hepatic steatosis in high-fat diet-fed rodent models (Zhang et al., 2018a), while Lonicerae japonicae flos extracts lower the triglyceride levels in serum and liver tissues of an animal model of hyperlipidemia (Li et al., 2015), and Hoveniae semen exhibited protective properties against ALD by reducing oxidative stress and preventing lipid dysmetabolism *in vivo* (Meng et al., 2020). Siraitiae fructus has been hypothesized to protect against NAFLD development by inhibiting fat droplet formation in the liver (Zhang et al., 2018b). Based on previous clinical experiences and animal experiments, we combined the four botanical drugs to form LAH for the first time and used network pharmacology to predict the mechanism of action of LAH, providing a new direction for the mechanism study of TCM formulas.

HPLC analysis revealed that chlorogenic acid, puerarin, daidzin, 3,5-dicaffeoylquinic acid and daidzein are the major components in LAH. In our network pharmacology approach, we focus on the potential targets of these five components for NAFLD. The results of KEGG and GO enrichment analysis showed that the pathways were mainly enriched in cancer, oxidative stress, non-alcoholic fatty liver disease, AMPK signaling pathway and other signal pathways, which indicated that LAH may regulated fatty liver *via* AMPK signaling pathway. Puerarin, which was the most abundant component of LAH. Puerarin has more target proteins in the interaction network, it may be the key component of LAH that plays the role of hepatoprotective. Previous studies have reported that puerarin increased serum insulin and decreased fasting serum glucose levels in ob/ob mice and STZ-induced diabetic rats, which are related to the activation of AMPK and PI3K/Akt pathways (Zhou et al., 2014). Furthermore, puerarin has a protective effect on alcohol-induced liver injury which may be related to the inhibition of oxidative stress (Zhou et al., 2014). Many studies have shown that puerarin has a variety of biological activities, but there are few studies focusing on the pharmacological action of puerarin and the underlying mechanisms and its antioxidant effect in NAFLD. To investigate the underlying mechanism, we performed OA-induced hepatic steatosis in HepG2 cells as an *in vitro* model of NAFLD. The results showed that puerarin treatment significantly decreased OA-induced HepG2 cells lipid droplets accumulation. In addition, puerarin increased the protein expression of phosphorylated AMPK and decreased SREBP1 and FAS protein expression in OA-induced HepG2 cells. As reported, AMPK phosphorylation can regulate lipid and carbohydrate metabolism in liver cells, which blocks SREBP1 expression to reduce fatty acid synthesis (Zhang et al., 2017). The protective effect of LAH is likely attributed to its regulatory role in the AMPK-SREBP1 signaling pathway.

We investigated the mechanism involved in the hepatoprotective effects of LAH in HFD-induced mice. The characteristics of NAFLD induced by HFD are the presence of higher concentrations of blood sugar, cholesterol, and TG in the blood circulation, as well as higher concentrations of liver damage markers, such as AST, ALT and LDH. (Eisinger et al., 2014). AST and ALT are important indices related to liver function, and the abnormally increased serum levels of AST and ALT imply the occurrence of hepatotoxicity and liver injury, which are closely associated with hyperlipidemia and hepatic steatosis (Sachdev et al., 2006). LAH administration lowered HFD-induced high serum levels of AST and ALT, indicating that LAH could protect mice against HFD-induced liver damage. Accumulated evidence has demonstrated that LAH improves hyperglycemia and dyslipidemia in HFD-induced mice, as indicated by lower circulating concentrations of cholesterol, glucose, and TG. It also decreased both liver and adipose tissue weight, decreased lipid accumulation in the liver, and ameliorated NAFLD in HFD-induced obese mice. Hepatic steatosis is defined by the excessive accumulation of triglycerides, causing more than 5% of hepatocytes containing visible lipid droplets in either a micro- or macrovesicular pattern (Ress and Kaser, 2016). LAH was able to reduce lipid accumulation and fat vacuoles as visualized using H&E staining and Oil Red O staining of liver histological slices. This effect can be attributed to a reduction in triglyceride levels in the blood and liver tissues.

SREBP1 is the most important transcription factor regulating *de novo* lipogenesis in the liver, and it specifically regulates the expression of many key enzymes in the fatty acid biosynthesis pathway (Wang et al., 2017). AMPK suppresses various anabolic pathways, stimulates catabolic pathways, it is considered to be the main therapeutic target for the treatment of non-alcoholic fatty liver (Kim et al., 2016). In addition, AMPK also the upstream kinase of SREBP1 in the liver. Therefore, activation of AMPK leads to the suppression of SREBP1 and reduces hepatocyte adipogenesis (Juszczak et al., 2020). *In vivo* studies confirmed that LAH increased AMPK phosphorylation, ACC phosphorylation and inhibited SREBP1 expression compared to the HFD group. In addition, we found that LAH improves CPT1 expression. CPT-1 is responsible for the transport of activated fatty acids into the mitochondria for β-oxidation (Qu et al., 2016). We found that LAH improved NAFLD in HFD-induced mice by enhancing AMPK expression to suppress SREBP1 activity and increasing β-oxidation for lipolysis. As predicted by network pharmacology approach, LAH may exert therapeutic effects on NAFLD by regulating the AMPK signaling pathway and decrease fatty acid synthesis.

HFD induces excessive accumulation of TC, cholesterol, free fatty acids, and lipids, leading to the production of reactive oxygen species (ROS) in the liver and accelerating oxidative stress (Tan and Norhaizan, 2019). Oxidative stress plays a vital role in the pathogenesis of NAFLD from steatosis to NASH, fibrosis, and cirrhosis and therefore, enhancing the cellular antioxidant system may have beneficial effects on NAFLD. AMPK activity is implicated in cellular redox homeostasis and plays a critical role in maintaining cell integrity by the inhibition of ROS and promote defense against oxidative stress (Hinchy et al., 2018). Our data indicated that LAH could increase AMPK phosphorylation in HFD mice. In order to explore the antioxidant effect of LAH, we measured the activity of antioxidant enzymes. Glutathione (GSH), one of the most abundant low molecular weight non-protein thiols, modulates physiological levels of ROS and is involved in the cell’s oxidative stress response. Glutathione reductase (GR) is responsible for maintaining the supply of reduced glutathione, and glutathione peroxidase (GPX) catalyzes the detoxification of H_2_O_2_ and lipid peroxides by reduced glutathione (Panday et al., 2020). Therefore, the activity of these antioxidants can be used as indicators of antioxidant activity (Perlemuter et al., 2005). In this study, the antioxidant enzyme activity of liver tissue in the HFD group was significantly reduced compared with that in the ND group, suggesting that NAFLD increased oxidative stress and decreased antioxidant capacity. However, LAH administration enhanced antioxidant capacity by increasing GR, GPX, and GSH activities. In addition, SOD and catalase levels were increased by LAH administration. Through these results, we confirmed the inhibitory effect of LAH on fatty liver by improving the antioxidant effect of the liver. In our future study, the research direction will explore the antioxidant mechanism of LAH.

In conclusion, this study demonstrates that supplementation with LAH efficiently inhibits fatty liver development and hepatotoxicity in HFD-induced NAFLD and prevents abnormal lipid accumulation by regulating AMPK/ACC activation, increasing β-oxidation, and downregulating FAS and SREBP1. Moreover, LAH also showed antioxidant activities and improves hyperglycemia and dyslipidaemia in NAFLD mice. This experimental evidence indicates that LAH has a potential protective effect in the prevention and improvement of NAFLD.

## Data Availability

The original contributions presented in the study are included in the article/Supplementary Material, further inquiries can be directed to the corresponding author.

## Abbreviations

ALT, Alanine aminotransferase; CAT, Catalase; CHOL, Cholesterol; DMEM, Dulbecco’s Modified Eagle Medium; FBS, Fetal bovine serum; FFAs, Free fatty acids; GLU, Glucose; GO, Gene Ontology; GPx, Glutathione peroxidase; GR, Glutathione reductase; GSH, Glutathione; HFD, High-fat diet; HPLC, High-performance liquid chromatography; KEGG, Kyoto Encyclopedia of Genes and Genomes; LAH, Livsooth authentic herbal formula; NAFLD, Non-alcoholic fatty liver disease; NASH, Non-alcoholic steatohepatitis; ND, Chow diet; OA, Oleic acid; PBS, Phosphate-buffered saline; PVDF, Polyvinylidene difluoride; ROS, Reactive oxygen species; SOD, Superoxide dismutase; TCM, Traditional Chinese medicine.
