# Supplementary material for: Hepatoprotective effect of botanical drug formula on high-fat diet-induced non-alcoholic fatty liver disease by inhibiting lipogenesis and promoting anti-oxidation
Source: Front Pharmacol. 2022 Nov 24;13:1026912. doi: 10.3389/fphar.2022.1026912 (PMC9729544; doi:10.3389/fphar.2022.1026912)
Supplement: Supplementary file 2 [file Table1.DOCX]

Supplementary Material

**Supplementary Table S1.** Components from the 4 herbs and their potential targets

| **Number** | **Component** | **Herb** | **Targets** |
| --- | --- | --- | --- |
| 1 | puerarin | *Pueraria lobata* | ABCB1, ACACA, ACACB, ACADM, ACE2, ACHE, ACLY, ACOX1, ACTA2, ADRP, AGTR1, AKR1B, AKT1, ALB, ALDH1A2, ALT1, AP1, AQP1, AQP3, AR, BAD, BAX, BCL2, BIRC5, BMP2B, CA2, CAMTA3, CASP3, CASP8, CASP9, CAT, CCN2, CDH1, CDKN1B, CDKN2A, CHUK, COL1A1, COL3A1, COX1, COX2, COX3, CPT1A, CREB1, CYGB, CYP19A1, CYP2E1, CYP7A1, DSG3, EGF, EIF4EBP1, ESR1, ESR2, F2R, FASLG, FASN, FBN1, FBN2, FGF1, FOS, FYN, GCLC, GDNF, GPER1, GPI, GPT, GSK3B, GSR, GSTP1, HAS1, HIF1AN, HK1, HMGCR, HMOX1, HSPB1, ICAM1, IFNA13, IFNG, IFNGR2, IGF1, IL1A, IL1B, IL6, INSR, ITGB5, JAK3, KL, LDHA, LDLR, LEPR, LIF, MAPK1, MAPK3, MAPK8, MAPK9, MC1R, MMP2, MMP9, MTOR, MYC, NFE2L2, NFKB1, NFKBIA, NOS1, NOS2, NOS3, NQO1, PARP1, PDGFA, PFKP, PGAM1, PGP, PIM1, PKC, PLAT, PLOD3, PML, PPARA, PPARG, PPARGC1A, PRKCD, PTGER1, PTGS2, PTPN1, RARA, REL, RPS6KB1, SELPLG, SIRT1, SLC25A4, SLC25A5, SLC2A1, SOD1, SOD3, SQSTM1, SRD5A2, SREBF1, STAT3, TBX1, TERT, TGFB1, TIMP1, TIMP2, TLR4, TNF, TPT1, TXN, TYRP1, UCP2, UGT1A1, UGT1A10, UGT1A3, UGT1A6, UGT1A7, UGT1A8, UGT1A9, VCAM1, VEGFA, XIAP |
| 2 | daidzin | *Pueraria lobata* | ALDH1B1, ACECD26, CAMTA3, F2, NCOA4, PTGS2, GPER1, PPARG, F10, F7, KDR, GSK3B, MAOA |
| 3 | daidzein | *Pueraria lobata* | COX1, GPER1, PPARG, COX2, NR2B1, ADRB2, HSP90AA1, MAPK 14, CHEK1, CDK1, PRKACA, RELA, VEGFA, FOS, CDKN1B, Eif3l, BAX, TNFRSF1A, FOS, IL6R, NOS2, CASP3, TP53, LRP1, CATB, IGFLR, STAT1, PPARG, CYP3A4, CAV1, ICAM1, MTTP, APOB, VCAM1, MSD1, NOS3,ECE1, GABBR1, IL4, CPT1A, CAMTA3, AHR, RHOA, MT2A, FCER2, MKI67, EP300, ATP5F1B, IGF1, HSD3B2, GH1, B4GALT1, GHRHR, CD5L, PDIL2-1, LARS1, CYP21A2, GADD45A, RAD51B |
| 4 | Chlorogenic acid | *Lonicera japonica* | ABCC1, ABCC2, ACHE, ACLY, ACTA2, ALB, ALPI, AQP1, AQP3, AR, BAMBI, BAX, BCL2, CASP3, CASP8, CASP9, CAT, CCL2, CCND1, CD44, CDH1, COL1A1, COL2A1, COMT, COX1, COX2, CS, CXCL1, CXCL8, CYGB, CYP19A1, CYP2E1, DSG3, EGF, ESR1, ESR2, F10, F2, FASN, FBN1, FBN2, FGF1, FSHB, FSHR, GABRA1, GABRB1, GCLC, GCLM, GPT, GPX1, GRN, GSR, GSTA1, HAS1, HLCS, HMGCR, HMOX1, HSPB1, IL1A, IL1B, IL6, ITGB2, JUN, KRT5, LCAT, LHB, LPL, LPO, LYZ, MAOA, MAOB, MAP2K4, MAPK1, MAPK14, MAPK3, MC1R, MGST1, MPO, MT1, MYD88, NFE2L2, NFKBIA, NFKBIB, NOS2, NOS3, NQO1, OCLN, PCNA, PECAM1, PGR, PIK3R1, PLOD3, PPARG, PPP2R1A, PPP5C, PTGS2, RAD23A, RARA, SELL, SLC11A2, SLC22A2, SLC2A4, SOD2, SOD3, SQSTM1, SRD5A2, STAR, TJP1, TLR3, TLR4, TNF, TPT1, TRP53, TXN, TYR, TYRP1, UGT1A10, UGT1A3, UGT1A7, UGT1A8, XDH |
| 5 | 3,5-Dicaffeoylquinic acid | *Lonicera japonica* | IL1B, IL6, MAPK1, MAPK3, MAPK9, NOS2, PTGS2, RELA, TNF |
